# Phase I study of dose‐escalated stereotactic body radiation therapy for locally advanced pancreatic head cancers: Initial clinical results

**DOI:** 10.1002/cam4.4214

**Published:** 2021-08-18

**Authors:** Shuiwang Qing, Lei Gu, Huojun Zhang

**Affiliations:** ^1^ Department of Radiation Oncology The First Affiliated Hospital of Naval Military Medical University Shanghai China

**Keywords:** DLT, dose‐escalation, GI toxicity, pancreatic head cancer, SBRT

## Abstract

**Purpose:**

To establish the maximum tolerated dose (MTD) of stereotactic body radiation therapy (SBRT) for locally advanced pancreatic head cancers.

**Methods:**

A total of 16 patients were included in the single‐institution phase I dose‐escalation study. The initial dose level was 35 Gy in five fractions, doses were then sequentially escalated to 37.5 Gy, 40 Gy, 42.5 Gy, and 45 Gy. The dose‐limiting toxicity (DLT) was defined as III/IV GI (gastrointestinal) toxicity.

**Results:**

A total of 16 patients with locally advanced pancreatic head cancers were analyzed, 14 patients had received gemcitabine or S1‐based chemotherapy. Median OS and LPFS were 14.5 months and 12.5 months, respectively; The OS rates at 1 and 2 years were 68.8% and 25%, respectively. No grade 3 or 4 acute or late GI toxicities were observed. Grade 3 toxicities were observed in four patients with three hematologic toxicities and one biliary obstruction for acute toxicities, G1–2 of GI late toxicity were in 31.25% of patients.

**Conclusions:**

SBRT doses ranging from 35 to 45 Gy in five fractions could be given for patients with locally advanced pancreatic head cancers without severe GI toxicities, whereas the side effect of biliary obstruction should be paid more attention.

**Trial registration:**

Clinical trials:NCT02716207.

## Graphical abstract

This is the first study for dose escalating of SBRT in pancreas head cancers.This research showed SBRT doses ranging from 35 to 45 Gy in five fractions could be given for patients with locally advanced pancreatic head cancers without severe GI toxicities, whereas the side effect of biliary obstruction should be paid more attention.

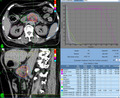

## 1 INTRODUCTION

Pancreatic cancer (PC) is the seventh leading cause of cancer mortality among both genders from Cancer Statistics 2019. Approximately 80% of patients developed with metastatic or locally advanced disease at the time of diagnosis.^1^, ^2^, ^3^ The median survival for patients with unresectable locally advanced pancreatic cancer (LAPC) who receive chemoradiotherapy was 11–15 months.^4^, ^5^, ^6^ However, the role of radiation for LAPC was controversial, chemoradiation therapy is still considered as one of the effective treatment options for LAPC in NCCN guidelines.^7^

The pancreas is anatomically close to the stomach and duodenum. With the advancement in radiotherapy, compared with conventional fractionated radiotherapy, SBRT has dramatically improved the survival benefits by offering excellent local control by delivering a conformal higher biologically effective dose (BED) and a minimal dose to the surrounding critical tissues.

A systematic review analyzed^8^ the outcome from 19 trials of SBRT for LAPC patients, the median OS was 17 months (5.7–47 months), 1‐year OS was 51.6%, and 1‐year local control rate was 72.3%. The reported rates of acute or late adverse events were generally tolerated with the occurrence of G3–4 gastrointestinal toxicities less than 10%. The doses were delivered ranged from 18 to 50 Gy in 1–8 fractions.

Although SBRT showed several advantages compared to conventional fractionated radiotherapy for LAPC, there are limited published prospective studies about contouring and dose escalating for pancreas SBRT especially pancreas head cancers.

We proposed to conduct a prospective serial dose‐escalation study for patients with LAPC to determine the optimal SBRT dose of CyberKnife SBRT based on a five fractions treatment regimen. The primary endpoint of this phase I trial was the incidence of acute and late gastrointestinal (GI) toxicity outcomes, secondary endpoints included overall survival (OS), clinical response rate, progression‐free survival (PFS), and the pain relief by NRS scores.

## 2 MATERIALS AND METHODS

### 2.1 Eligibility

The protocol of inclusion criteria and radiation treatment planning were based on our previous publication (NCT02716207), inclusion criteria: tumor size <5 cm, locally advanced unresectable pancreatic head cancer, exclusion criteria: distance between tumor and critical organ is less than 5 mm, accepted previous treatment (surgery, chemotherapy or radiotherapy) for pancreatic tumor.^9^ From September 2016 to September 2018, all 16 patients got the pathological diagnosis by biopsy with the tumors located in the head of pancreas. The patients were offered SBRT treatment delivered by CyberKnife (Accuray). Tumor staging was based on NCCN guidelines (2021), 8^th^ AJCC (American Joint Committee on cancer) TNM staging of pancreatic cancer.^7^ This prospective study obtained the approval from the independent Ethics Committee of Changhai hospital, the ethics number is 2016–030–01.

### 2.2 Treatment technique and patients’ follow‐up

Patients were placed in the supine position using whole‐body vacuum pad and underwent planning CT with a slice thickness of 3 mm. The normal organs at risk included duodenum, stomach, small intestine, liver, spinal cord, and kidney. The definitions of gross tumor volume (GTV), clinical target volume (CTV), and planning target volume (PTV) were based on the protocol. GTV is defined as the visible tumor based on enhanced CT. CTV equals the GTV. PTV was usually defined as the region of 2–5 mm outside of CTV.^9^

All patients were treated with CyberKnife platform with respiratory tracking as well as fiducial tracking system by placing one gold fiducial (CT‐guided) which is 0.9 mm in diameter and 3 mm in length (CIVCO, USA) close to the tumor. Treatment planning CTs were performed at least 7 days after fiducial placement. All patients were with follow‐up at regular intervals (every 3 month within a year) to estimate the tumor size and the presence of symptoms by enhancement abdomen CT scan.

### 2.3 Chemotherapy

Patients received 4–6 cycles adjuvant chemotherapy with gemcitabine based and S‐1 in 1 month after SBRT. Concurrent S‐1 of 80 mg/m^2^ was given twice a day on the commencement of radiotherapy, for 28 days followed by a 14‐day rest. Gemcitabine was given with 1000 mg/m^2^ on days 1, 8 during each 3‐week cycle.

### 2.4 Formulas and statistical analysis

The biological effective dose (BED) was calculated according to the following formula, *BED* = nd*[1+*d*/*(α*/*β)]*. (*d* is the dose per fraction and *n* is the number of fractions and a value of 10 was used for the *α*/*β*‐ratio). OS started with the first day of radiotherapy. Progression‐free survival was calculated from the date of radiotherapy to the date of the event or last follow‐up. OS and PFS were assessed with the Kaplan–Meier method. Statistical significance was set at *p* < 0.05.

## 3 RESULTS

### 3.1 Patient characteristics

Sixteen patients were enrolled in this study, 15 of them who received SBRT died. Patient characteristics are summarized in Table 1. The median age at the time of diagnosis was 65 years (range 44–72 years). A total of 14 patients (86.7%) received chemotherapy after SBRT, 56.3% of patients were male. Four patients were treated at dose level 1 (35 Gy/5F), and three at other dose levels, respectively. The median planning target volume was 27.3 cc (range 11.6–48.3 cc).

**TABLE 1:** Patient information

Pt Number	Age/sex	Tumor diameter(cm)	Stage	Chemotherapy after SBRT	TD(Gy)/fx	CA199(U/ml)	Pain (NRS)	Histology
1	44/M	4.1	T3N0	Gem based	35/5	>1200	7	Adenocarcinoma
2	63/F	3.9	T4N0	None	35/5	2	7	Adenocarcinoma
3	50/M	3	T2N1	Gem based	35/5	>1200	5	Adenocarcinoma
4	70/F	3.5	T4N0	S1	35/5	23	7	Adenocarcinoma
5	66/F	3.4	T4N0	Gem based	37.5/5	>1200	7	Adenocarcinoma
6	71/F	4.6	T3N0	Gem+S1	37.5/5	25.5	8	Adenocarcinoma
7	71/F	3.5	T4N0	S1	37.5/5	105	6	Unclassified
8	72/M	5	T3N0	Gem based	40/5	>1200	3	Adenocarcinoma
9	63/M	2.3	T2N1	S1	40/5	125	3	Unclassified
10	61/M	4.2	T3N0	S1	40/5	123.4	5	Adenocarcinoma
11	67/F	5	T3N1	S1	42.5/5	>1200	8	Adenocarcinoma
12	65/M	4.4	T3N0	S1	42.5/5	2	5	Adenocarcinoma
13	64/M	4.8	T3N1	Gem+S1	42.5/5	>1200	9	Adenocarcinoma
14	65/F	3.2	T2N1	S1	45/5	313	5	Unclassified
15	68/M	3.2	T4N0	Gem based	45/5	381	8	Unclassified
16	65/M	3.4	T4N0	None	45/5	89	7	Adenocarcinoma

### 3.2 Tumor response and survival

Best response was evaluated by response evaluation criteria in solid tumors (RECIST) criteria in all 16 patients enrolled (Table 2). The ORR was 31.25% (5/16). A total of two patients achieved complete responses, one of the responses were in level 2 (37.5 Gy/5F), and one response occurred in level 4 (42.5 Gy/5F). Three patients had partial response as the best response, one of partial response was in level 2 (37.5 Gy/5F), and two were in level 5 (45 Gy/5F).

**TABLE 2:** Patient treatment characteristics (n = 16)

	Number of patients (%)
Dose per fraction/total dose 7 Gy/35 Gy	4 (25)
7.5 Gy/37.5 Gy	3 (18.75)
8 Gy/40 Gy	3 (18.75)
8.5 Gy/42.5 Gy	3 (18.75)
9 Gy/45 Gy	3 (18.75)
PTV volume	Median 27.3 cc (range 11.6–48.3 cc)
Max dose to duodenum (5 cc)	Median 19.9 Gy (range 9–21.8 Gy)
Max dose to stomach (10 cc)	Median 21.6 Gy (range 14.8–28 Gy)
Max dose to bowel (5 cc)	Median 20.81 Gy (range 11–26.1 Gy)

The median follow‐up time was 15 months when measured from SBRT (9.3–45 months).

**FIGURE 1:**
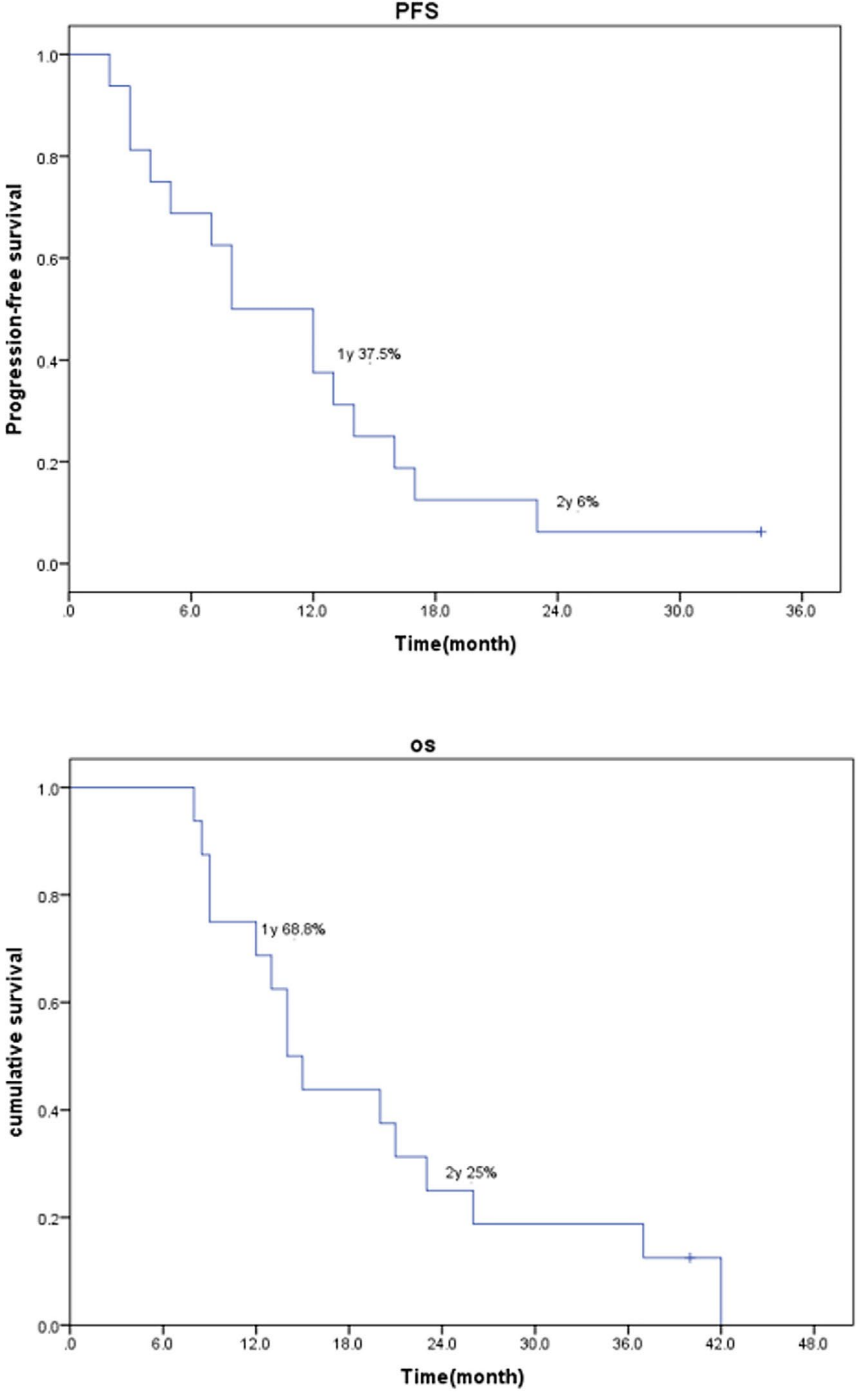
Kaplan–Meier estimates of progression‐free survival and overall survival for all 16 patients

**FIGURE 2:**
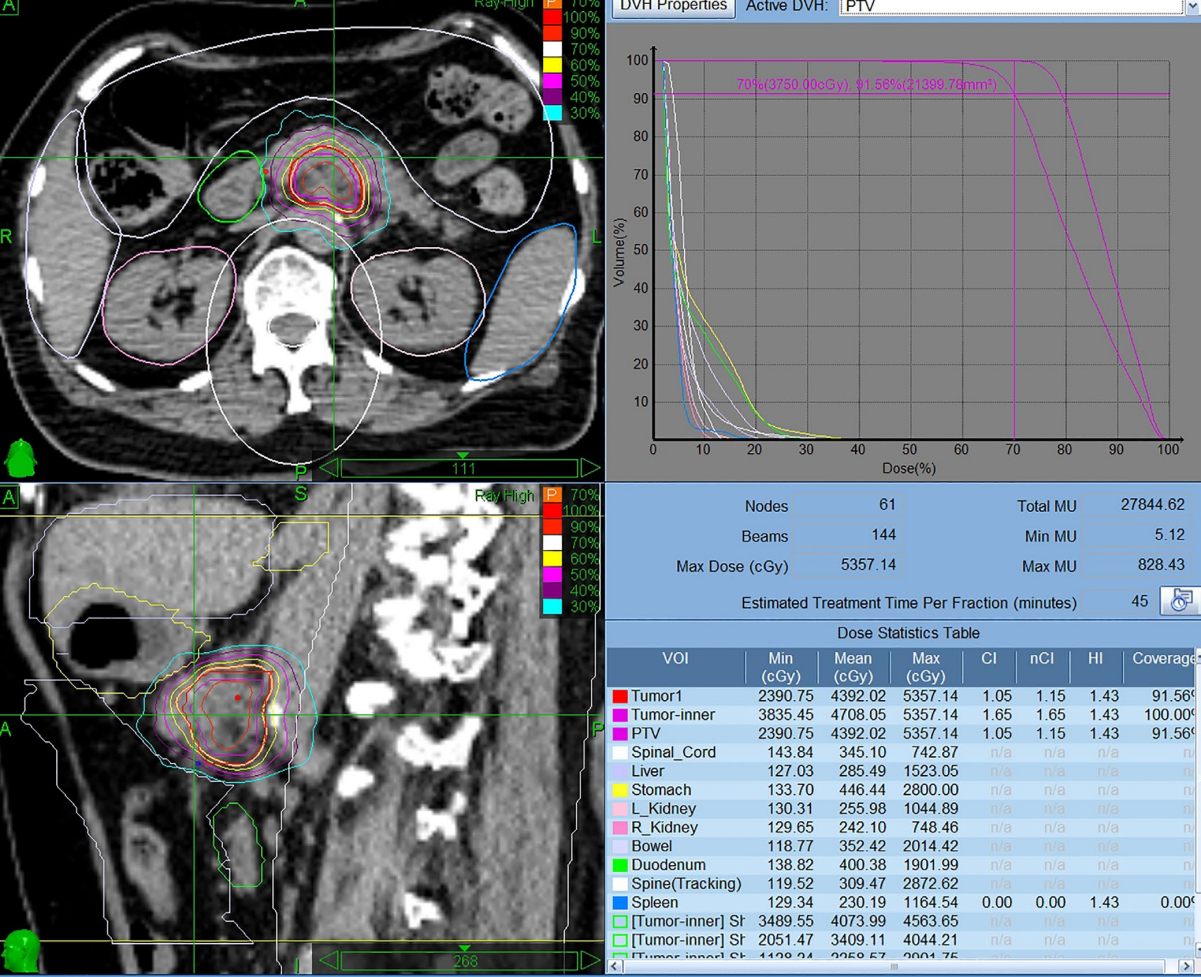
An accumulated dose distribution of radiation therapy for case 7 of locally advanced pancreatic cancer during the first SBRT course

**FIGURE 3:**
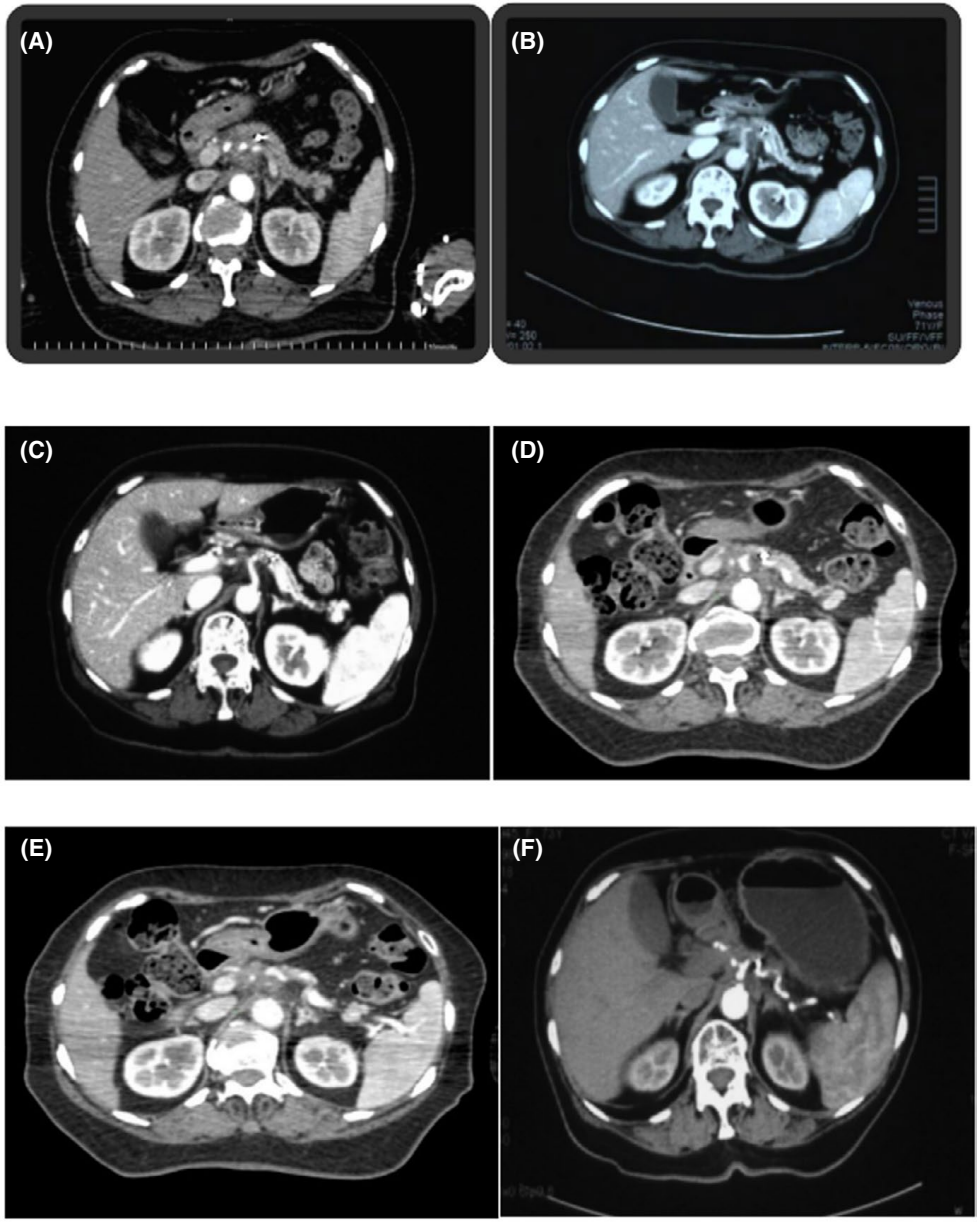
A. CT scan prior to the first‐course treatment shows a mass in the pancreatic head. B, C. CT scan 3 months and 9 months following the first SBRT course. D. CT scan prior to the second‐course treatment shows the local recurrence of the tumor in the pancreatic head. E. CT scan 1 months following the second SBRT course. F. CT scan 6 months following the second SBRT course

Median overall survival was 14.5 months (8–42 months). Median progression‐free survival was 10 months (2–34 months). Median local progression‐free survival was 12.5 months (8–34 months) Figure 1. The OS rates at 1 and 2 years were 68.8% and 25%, respectively. The PFS rates at 1 and 2 years were 37.5% and 6%, respectively (Table 3). The LPFS rate at 1 year was 69%. Kaplan–Meier curves for both PFS and OS are shown in Figure 1. Eight patients died of distant metastases (liver, bone, and multiple metastases), four died of local recurrences, one died of both local recurrence and distant metastasis, and two died of malignant ascites and dyscrasia. Two patients accepted reirradiation of second‐course SBRT (case 7 and case 12) in the 17th months and 34th months followed by the first‐course SBRT, respectively (Figures 2 and 3).

**TABLE 3:** Post‐treatment evaluation

Pt Number	The best response	CA199 (U/ml)	Pain(NRS)	OS(m)	PFS(m)
1	SD	>1200	3	9	3
2	SD	2	5	8.5	4
3	SD	352.6	3	42	23
4	PD	28	2	8	2
5	PR	48	1	21	14
6	SD	25.5	3	23	13
7	CR	24	2	37	17
8	SD	>1000	3	14	8
9	SD	70	3	13	5
10	SD	123.4	2	12	7
11	SD	>1200	5	9	3
12	CR	19	2	40+	34
13	SD	>1200	6	20	12
14	PR	29	3	26	16
15	SD	244	5	14	8
16	PR	22.36	2	15	12

The relief of abdominal or back pain was reviewed by numerical rating scale (NRS) scoring evaluation. The pain was obviously complained by 14 enrolled patients assessed by NRS system with the range from 5 to 9 scores prior to radiation therapy. Opioids had to be prescribed to eight patients to control the pain. The pain relief rate was up to 100% for patients with NRS above 5 scores. In the meanwhile, the medication was reduced for six patients.

### 3.3 Toxicities

Treatment‐related toxicities are summarized in Table 3. The most common acute toxicities were hematologic toxicities (G1 31%, G2 18.75%, and G3 18.75%), fatigue (G1 37.5% and G2 6.25%), nausea (G1 25% and G2 12.5%), abdominal pain (G1 12.5%), biliary obstruction (G1 12.5% and G3 6.25%), and diarrhea (G1 6.25%). Grade 3 toxicities were observed in four patients with three grade 3 hematologic toxicities and one grade 3 biliary obstruction who accepted endoscopic enteral stenting. For late chemoradiation‐related toxicity, three patients complained grade one gastrointestinal toxicities, two patients for G2. G3 biliary obstruction was observed in one patient (6.25%). No other G3 or greater GI complications were observed (Table 4).

**TABLE 4:** Toxicity summary

Toxicity		CTCAE v4.0 Grade		
		1	2	3
Acute	Hematologic	5	3	3
	Nausea	4	2	0
	Anorexia	2	0	0
	Diarrhea	1	0	0
	Abdominal pain	2	0	0
	Fatigue	6	1	0
	Biliary obstruction	2	0	1
Late	Gastrointestinal	3	2	0
	Biliary obstruction	0	0	1

Grade 1 biliary obstruction patient experienced transient hyperbilirubinemia (total bilirubin ≥3.0 mg/dL or direct bilirubin ≥1.5 mg/dL) during or after stereotactic body radiation therapy.

## 4 DISCUSSION

Pancreatic ductal adenocarcinoma has been regarded as radio‐resistant tumor, a phase III trial found there were any significant survival benefits for conventional radiotherapy plus chemotherapy over chemotherapy alone for LAPC patients.^10^ Although SBRT is a relatively new treatment option for LAPC, the high reported rates of tumor control and low toxicity encourage us to conduct this study.

According to the limited prospective evidence by the time of the study, there is insufficient evidence to recommend a consensus for the dose‐escalation of SBRT, optimal technique, or delivery system. In our study, a stepwise dose‐escalation phase I trial was done and 45 Gy in five fractions was reached without DLTs.

The first phase I dose‐escalation trial on SBRT for LAPC was investigated at Stanford University where the radiation dose was successfully delivered up to 25 Gy in a single fraction without grade ≥3 toxicities.^11^ Subsequent studies of SBRT with single fraction showed 94%–100% local control at 1 year and 20%–44% grade 2 or higher late GI toxicities, including duodenal ulcers, stenosis, or perforation.^11^, ^12^, ^13^, ^14^

Several retrospective studies found a multi‐fraction approach of 5 fractions can potentially decrease the risk of late toxicities compared to 1–3 fractions commonly used in other institutions.^15^, ^16^ Compared to prior studies using single‐fraction SBRT, the fractioned SBRT reduced grade 2 or higher acute GI toxicities to 2%, and grade 2 or higher late toxicity to 11%.^5^

In this study, 25% of patients experienced grade 3 acute SBRT‐related toxicities, three with hematologic toxicities, and one with biliary obstruction. Moreover, one patient developed G3 biliary obstruction for late chemoradiation‐related toxicity. No grade 3 or higher acute and later GI toxicities were observed.

The objective response rate of 31.25% and the median OS of 14.5 months observed in this trial are similar to the median OS of 10.6–20.0 months in prior fractionated SBRT studies.^4^, ^17^, ^18^ Moreover, a systematic review including 1009 LAPC patients in 19 studies treated with SBRT, reported the median OS of 17 months and 72.3% 1‐year LC.^8^ The 69% of 1‐year LPFS observed here was close to 1‐year LPFS rates of 70%–87% seen in several studies of fractionated SBRT.^4^, ^17^, ^18^

Arcelli A^19^ reported a positive impact of SBRT BED_10_ ≥ 48 Gy which was correlated with both LC and OS through multivariate analysis. Additionally, our previous study^20^ showed that patients can get a better OS with BED_10_ ≥60 Gy, and Krishnan^21^ also reported that BED_10_ > 70 Gy was a positive predictor of better OS as well as better local PFS. In our study, an escalation prescription dose from 35 to 45 Gy in five fractions was administered (BED_10_ range: 59.5–85.5 Gy), which was believed to be safe and effective dose for LAPC by most investigators.

Pancreatic adenocarcinoma (PC) can occur in any part of the pancreas, whereas nearly 75%–80% patient with PC are found in the head of the pancreas.^22^ This is the first study on dose‐escalated SBRT for locally advanced pancreatic head cancers. Several studies suggested that there were differences between pancreatic head cancer and pancreatic body/tail cancer in survival prognosis and treatment.^23^, ^24^, ^25^ As for treatment of radiation therapy, the anatomical tumor location plays an important role for the treatment of LAPC. Pancreatic lesions were located in deep intraperitoneal cavity especially pancreatic head cancer, which was adjacent to critical organs such as duodenum, stomach, bowel, liver, and kidney.

Courtney and his co‐worker have conducted a phase I study and reported a late grade 3 gastrointestinal (GI) toxicity rate of 6.7% in 45 Gy/5F dose group, whereas no severe late GI toxicity in 50 Gy/5F group. With the majority of pancreatic tumors arising in the head, neck, and uncinate process, the GI toxicities may depend not only on the total prescription dose but also the anatomy of the tumor position.^17^

SBRT could deliver ablative doses of radiation to the tumor volume with minimal margin, which can bring better clinical benefit and less toxicity for pancreatic head cancer. In our study, the G1 and G3 toxicities of biliary obstruction were observed in four patients, but no G3 or greater GI complications were noticed. It is known that patients with pancreatic head cancer mostly present jaundice due to the obstruction of the common bile duct during the course of disease development. As mentioned in NCCN guideline,^7^ endoscopic retrograde cholangiopancreatography (ERCP) with stent placement is a preferred recommendation prior to initiation of RT. And patients with grade 3 biliary obstruction underwent endoscopic enteral stenting in our study. Since no patients had suffered obstruction symptoms prior to SBRT, it is not clear whether biliary obstruction was related to survival, however the poor OS was observed in patients with severe biliary obstruction. Except for the severe GI toxicities, we should pay more attention to the toxicity of biliary obstruction in pancreatic head cancers during SBRT treatment.

Although our phase I study had not met its primary endpoint of establishing the MTD for SBRT in LAPC treatment, a stepwise dose‐escalation to 45 Gy in five fractions was reached without DLT of GI toxicity. The further prospective randomized studies would be required to define the efficacy and toxicity of SBRT in patients with locally advanced pancreatic head cancers.

In conclusion, SBRT has being used to treat patients with multiple inoperable cancers. SBRT doses ranging from 35 to 45 Gy in five fractions for patients with locally advanced pancreatic head cancers resulted in favorable FFLP and OS without severe GI toxicities.

## 5 ETHICAL APPROVAL STATEMENT

This prospective study obtained the approval from the independent Ethics Committee of Changhai hospital and all patients had signed informed consents.

## CONFLICT OF INTEREST

The author(s) declare no competing interests.

## AUTHORS’ CONTRIBUTIONS

SQ and LG have made equal contribution to the study and drafted the manuscript. ZHJ is the principle investigator (PI) of this study and made contributions to the study design.

## Data Availability

All datasets generated for this study are available from the corresponding author.
